# The Thumb and the Brain

**Published:** 1898-09

**Authors:** 


### The Thumb and the Brain.

Dr. Burton Ward declares there is one infallible symptom indicating whether one is sane or not. Let a person speak ever so rationally and act ever so sedately, if his or her thumbs remain inactive there is no doubt of insanity. Lunatics seldom make use of their thumbs when writing, drawing or saluting.—*N. Y. Med. Record.*

### Bone Grafting.

Bardenhaer has succeeded in making a bone transplantation a substitute for amputation.

